# Enhancing the Electrochemical Performance of SbTe Bimetallic Anodes for High-Performance Sodium-Ion Batteries: Roles of the Binder and Carbon Support Matrix

**DOI:** 10.3390/nano9081134

**Published:** 2019-08-07

**Authors:** Vijay Mohan Nagulapati, Doo Soo Kim, Jinwoo Oh, Jin Hong Lee, Jaehyun Hur, Il Tae Kim, Seung Geol Lee

**Affiliations:** 1 Department of Organic Material Science and Engineering, Pusan National University, 2, Busandaehak-ro 63beon gil, Geumjeong-gu, Busan 46241, Korea; 2 Department of Chemical and Biological Engineering, Gachon University, Seongnam-si, Gyeonggido 13120, Korea; 3 Photo-electronic Hybrids Research Center, Korea Institute of Science and Technology, 5 Hwarang-ro 14-gil, Seongbuk Gu, Seoul 02729, Korea

**Keywords:** sodium-ion battery, PAA binder, bimetallic anode, antimony, tellurium

## Abstract

Synergism between the alloy materials and the carbon support matrix, in conjunction with the binder and electrolyte additives, is of utmost importance when developing sodium-ion batteries as viable replacements for lithium-ion batteries. In this study, we demonstrate the importance of the binder and carbon support matrix in enhancing the stabilities, cyclabilities, and capacity retentions of bimetallic anodes in sodium-ion batteries. SbTe electrodes containing 20%, 30%, and 40% carbon were fabricated with polyvinylidene fluoride (PVDF) and polyacrylic acid (PAA) binders, and electrochemically evaluated at a current rate of 100 mA g^−1^ using electrolytes with 0%, 2%, and 5% added fluoroethylene carbonate (FEC). The electrodes with the PVDF binder in cells with 5% FEC added to the electrolyte showed capacity retentions that increased with increasing carbon percentage, delivering reversible capacities of 34, 69, and 168 mAh g^−1^ with 20%, 30%, and 40% carbon; these electrodes retained 8.1%, 17.4%, and 44.8% of their respective capacities after 100 cycles. However, electrodes composed of the PAA binder in cells with 5% FEC added to the electrolyte delivered reversible capacities of 408, 373, and 341 mAh g^−1^ with 20%, 30%, and 40% carbon; 93.5%, 93.4%, and 94.4% of their respective capacities were retained after 100 cycles. The carbon support matrix plays a significant role in improving the stability, cyclability, and capacity retention of the electrode. However, when the tradeoff between capacity and cyclability associated with carbon percentage is considered, the binder plays a significantly more prominent role in achieving high capacities, high cyclabilities, and enhanced retention rates.

## 1. Introduction

Sodium-ion batteries (SIBs) are being pursued with renewed enthusiasm owing to the growing global demand for alternative energy-storage devices and increasing concerns about the sustainability of lithium supplies [1]. In the search for a more sustainable alternative, sodium-ion batteries have garnered increased interest due to the abundance, low cost, and wide geographical distribution of sodium [2]. Sodium has similar chemical properties to lithium, which enables the knowledge and experience acquired from research into lithium-ion batteries (LIBs) to be applied directly to SIBs [3,4,5,6,7,8,9]. In addition, sodium has a very suitable low redox potential of −2.71 V vs. SHE (standard hydrogen electrode), which is only 0.3 V more positive than that of lithium (−3.04 V vs. SHE) [8,10]. However, SIBs still need to overcome hurdles that include low energy densities, inferior cycling stabilities, and the very large volume changes associated with sodiation/desodiation, which is due to the large radius of Na^+^ (1.02 Å) [11,12,13]. Various approaches have been proposed for overcoming these hurdles, of which bimetallic alloy systems as anodes are considered to be the most viable. Bimetallic systems with intermetallic components provide buffering phases that accommodate volume changes during cycling [14,15,16]. Antimony-based materials are proving to be among promising alloying-type materials for SIBs owing to their high theoretical capacities with Na (Na_3_Sb = 660 mAh g^−1^) [17,18]. However, antimony suffers from a large volume change (~390%) which results in rapid capacity decay [19,20]. Tellurium is gaining significant attention because of the sodium-active nature of group VI elements. Te has a high electronic conductivity (~2 × 10^2^ S cm^−1^) and a high density (6.24 g cm^−3^) that provide a high theoretical volumetric capacity of 2621 mAh cm^−3^ [21]. Bimetallic SbTe is a promising anode material for use in SIBs, as Sb and Te react with Na at different potentials, which reduces the overall volume change. In addition, more interest has recently been given to the volumetric charge capacity of a battery than the charge-to-mass ratio alone, and antimony and tellurium have specific densities of 6.50 and 6.24 g cm^−3^, respectively. Sb_2_Te_3_ has been reported to have a density of 6.66 g cm^−3^ and a volumetric capacity of 3419 mAh cm^−3^ [22,23]; hence, SbTe bimetallic compounds have been the focus of much attention. 

Another approach involves the addition of a conductive matrix that encapsulates the active material and provides conductive pathways for electrons and Na^+^ ions while accommodating volume expansions [24,25]. The use of carbon (acetylene black) as a conductive buffer matrix is an effective approach due to its excellent electrical conductivity, electrochemical stability, compressibility, and low cost [11,26]. The use of functional binders is also being studied as a viable way of improving the electrochemical performance of electrodes. Functional binders mitigate against the adverse effects of desodiation/sodiation by forming covalent bonds between the surface –OH groups of the active material, current collector, and the –COOH groups of the binder, resulting in enhanced adhesion capabilities and increased binding strengths. In addition, they keep the electrode intact by adhering to the current collector. Polyacrylic acid (PAA) is a functional polymeric binder with carboxylic acid functional groups (–COOH) that form weak hydrogen bonds with the surface –OH groups of the active material, resulting in a conductive ionic film on the surface of the active material [27,28,29]. Li et al. reported that its strong binding ability, the uniform distribution of the active material in electrode, and its suitable swelling properties are advantages of the PAA binder [30,31]. Polyvinylidene fluoride (PVDF) is a common binder used in LIBs; it is a linear homopolymer binder that is used in batteries for its strong adhesion and electrochemical stability. However, PVDF cannot sustain stable cycling in SIBs due to its low tensile strength, which is attributable to its high swellability [32]. In addition, Na^+^ reacts with PVDF to form NaF, thereby degrading the binder and leading to the loss of its adhesion capabilities and diminished electrochemical performance [33].

In this work, we synthesized SbTe bimetallic composites with three different concentrations (20%, 30%, and 40%) of the carbon buffer matrix by high-energy machine milling (HEMM). The SbTe compounds loaded with 20% carbon (SbTe-C20), 30% carbon (SbTe-C30), and 40% carbon (SbTe-C40) were electrochemically evaluated with PVDF and PAA binders, and 0%, 2%, and 5% fluoroethylene carbonate (FEC) as the electrolyte additive. The electrodes with the PAA binder and the electrolyte with 5% added FEC exhibited excellent reversible capacities and enhanced capacity retention capabilities even after 100 cycles, whereas the electrodes with the PVDF binder and the electrolyte with 5% added FEC showed capacity retentions that improved with increasing carbon percentage. Increasing the amount of the carbon support matrix has a definite positive effect on the stabilities, cyclabilities, and capacity retentions of these SbTe bimetallic composites. On the other hand, the use of the functional polymeric PAA binder has a much more profound and significant effect on the overall electrochemical performance of these bimetallic alloy electrodes.

## 2. Experimental

### 2.1. Preparing the SbTe-C Bimetallic Compounds

The SbTe-C bimetallic compounds were prepared using Sb (Aldrich, St. Louis, MI, USA, 100 mesh, 99.5%), Te (Aldrich, St. Louis, MI, USA, 200 mesh, 99.8%), and carbon (Alfa Aesar, Haverhill, MA, USA, 100% compressed, 99.9+%) as the raw materials. First, pristine Sb and Te were manually mixed in a mortar in a 1:1 molar ratio. Carbon (acetylene black) (20, 30, or 40 wt%) was then added and the mixture was subjected to high-energy mechanical milling by placing the as-prepared powder in a container with 1/2”- and 1/4”-diameter zirconia balls in a powder:ball weight ratio of 1:20. The container was then sealed in an argon-filled glove box and installed in a planetary ball mill (Pulverisette 5, Fritsch, Idar-Oberstein, Germany), which was operated at 300 rpm for 48 h at ambient temperature. The measured tap densities were 1.315 g/mL for SbTe-C20, 1.235 g/mL for SbTe-C30, and 1.141 g/mL for SbTe-C20, respectively.

### 2.2. Material Characterization

The active materials, prepared as described above, were characterized by powder X-ray diffractometry (XRD, Philips X’Pert-MPD, Malvern, UK) with Cu Kα radiation in the 5–80° 2ϴ range at a constant step angle and step size of 0.013° and 97.92 s, respectively. Scanning electron microscopy (SEM, Hitachi S3500N, Krefeld, Germany) was used to examine the surface morphologies, and SEM augmented with energy-dispersive X-ray spectroscopy (EDS, Hitachi S3500N, Krefeld, Germany) was used to create elemental maps that confirmed the homogeneous nature of the cast dispersion of the active material, as well as the approximate local Sb, Te, and C contents of the SbTe-C20, SbTe-C30, and SbTe-C40 composites.

### 2.3. Electrochemical Experiments

The active-material composites were converted into slurries consisting of 70% active material, 15% super P carbon (Alfa Aesar, Haverhill, MA, USA, 99+ %), and 15% PVDF (Sigma Aldrich, St. Louis, MI, USA, Mw = 534,000) or 15% PAA (Sigma Aldrich, St. Louis, MI, USA, Mw = 450,000). 1-Methyl-2-pyrrolidinone (NMP, Sigma Aldrich, St. Louis, MI, USA, >99.0%) was used as solvent and each slurry was mixed for 24 h. The viscosity of the slurry was maintained by adding an adequate amount of NMP while mixing. The doctor blade method was used to cast each slurry onto Cu foil, after which it was dried for 3 h at 70 °C in air oven and then at 80 °C in a vacuum oven for 24 h. The cast materials were cut into 12-mm-diameter electrodes for electrochemical testing. The active material loading (mass loading) was about 1.5–2.0 mg for electrodes with PVDF binder and 2.2–2.5 mg for electrodes with PAA binder. CR 2032 coin cells were assembled in an argon-filled glove box. Each coin cell contained a glass-fiber separator, with sodium metal as the counter electrode (APFA, Millipore, Burlington, MA, USA), and 2% or 5% FEC (TCI, >98.0%) and 1 M NaClO_4_ in 1:1 (v/v) ethylene carbonate (EC, Sigma Aldrich, St. Louis, MI, USA, anhydrous 99.7%)/propylene carbonate (PC, Sigma Aldrich, St. Louis, MI, USA, anhydrous, 99%) as the electrolyte. Galvanostatic discharge/charge experiments were performed using an automatic battery cycler (WonATech, WBCS3000, Seoul, South Korea) in the of 0.0–3.0 V (vs. Na/Na^+^) voltage range at a current rate of 100 mA g^−1^. Rate capabilities were examined by performing five discharge/charge cycles each at current rates of 100, 300, 500, 1000, 1500, and 2000 mA g^−1^, and then back to 100 mA g^−1^. EIS (WonATech, electrochemical impedance spectroscopy, Seoul, South Korea) was performed using a WonATech (WonATech, ZIVE SP1, Seoul, South Korea) workstation in the 100 kHz to 0.1 Hz frequency range at an applied-voltage amplitude of 10 mV.

## 3. Results and Discussion

### 3.1. Characterizing Structures and Morphologies

#### 3.1.1. XRD

**Figure 1:**
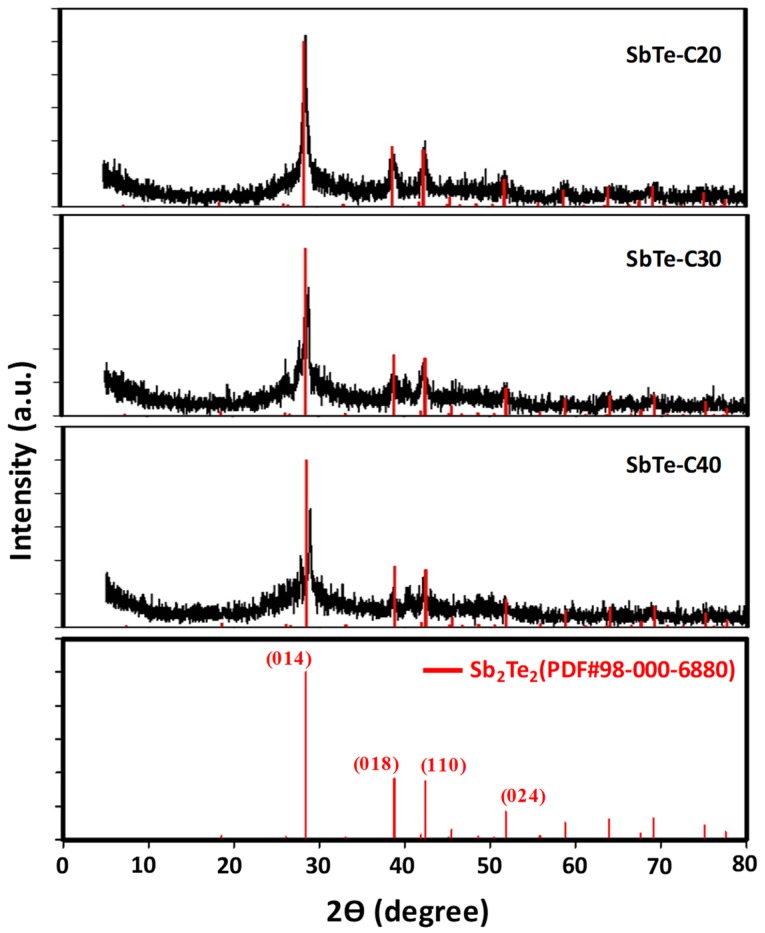
XRD patterns of the as-prepared SbTe-C20, SbTe-C30, SbTe-C40 composites.

The XRD patterns of the SbTe-C20, SbTe-C30, and SbTe-C40 powders produced by HEMM are shown in Figure 1, which reveal that a pure hexagonal SbTe phase was formed in each case [34]. All of the diffraction peaks observed for SbTe are in good agreement with those reported for Sb_2_Te_2_ (PDF# 98-000-6880), in which the prominent peaks at around 28.4°, 38.7°, 42.4°, and 51.8° are attributed to the (014), (018), (110), and (024) planes of SbTe. The intensities of the peaks were observed to decrease with increasing amorphous-carbon content, which is attributable to the formation of uniform coatings of amorphous carbon on the surfaces of the SbTe particles during HEMM. A small peak was observed at 40.27° in the XRD patterns of the compounds containing 30% and 40% carbon, which was more intense in the pattern of the latter. We assume that this peak is due to excess carbon present in the compound. The peak observed at 27° to the left of 014 peak is assumed to be due to phase overlap of hexagonal Sb_2_Te_2_ with that of the Te [34]. Each SbTe-C composite exhibited only an SbTe phase, which indicates that the alloying reaction was complete following HEMM.

#### 3.1.2. SEM and EDS

**Figure 2:**
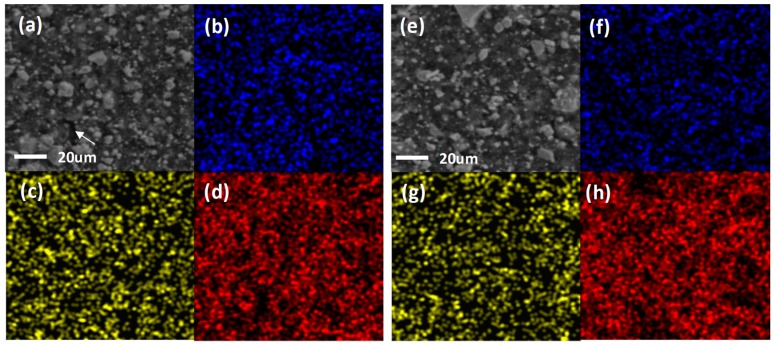
SEM images of the SbTe-C20 electrode prepared with (**a**) the PVDF binder and (**e**) the PAA binder. Sb, Te, and C EDS maps corresponding to: (**b**–**d**) panel a, and (**f**–**h**) panel e.

Figure 2, Figures S1 and S2 (Supporting Information) display SEM images and EDS elemental maps of the SbTe-C20, SbTe-C30, and SbTe-C40 electrodes prepared with the PVDF and PAA binders, respectively. The SEM image of each electrode shows irregularly shaped particles that are several micrometers in size. The SbTe-C20 electrode prepared with the PVDF binder (Figure 2a) exhibits small visible cracks on its surface, while the SbTe-C20 electrode prepared with the PAA binder shows no visible cracks. This is ascribable to the poor adhesion of the PVDF binder, which leads to the isolation of particles, the aggregation of the active material, and poor electrochemical performance. On the other hand, the SEM images of the SbTe-C30 (Figure S1) and SbTe-C40 (Figure S2) electrodes exhibit no noticeable morphological differences on their surfaces, which is due to the excess-carbon coating that prevents the aggregation of the active material and maintains contact and conductivity. Figure 2, Figure S1 and S2 (b–d and f–h) show the Sb, Te, and C EDS elemental maps of the SbTe-C20, SbTe-C30, and SbTe-C40 electrodes prepared with the PVDF and PAA binders, respectively. These elemental maps reveal that all elements are homogeneously mixed in each electrode; all maps show that the Sb, Te, and C in each electrode are uniformly distributed. There is no evidence of any segregated composite, which indicates that all composites were well mixed, and that the active materials were homogeneously distributed throughout the conductive carbon matrix.

### 3.2. Electrochemical Performance 

#### 3.2.1. Voltage Profiles

**Figure 3:**
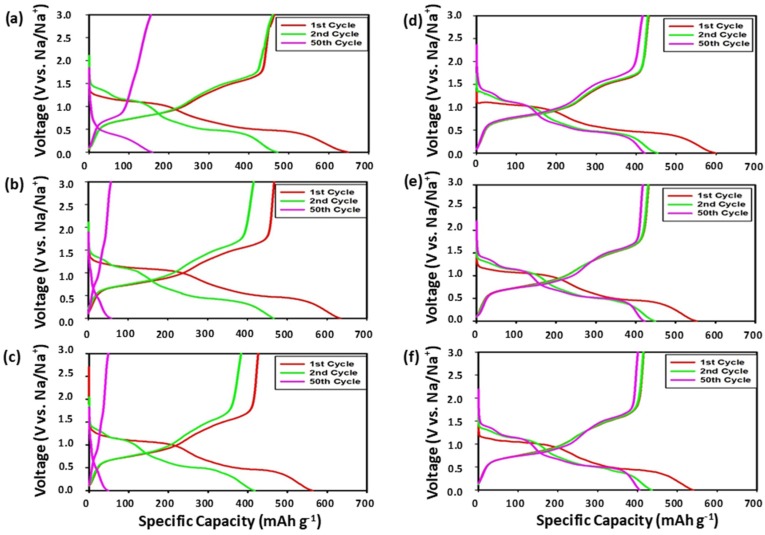
Voltage profiles of SbTe-C20 electrodes containing (**a**–**c**) 0%, 2%, and 5% FEC (respectively) and the PVDF binder, and (**d**–**f**) 0%, 2%, and 5% FEC (respectively) and the PAA binder.

**Figure 4:**
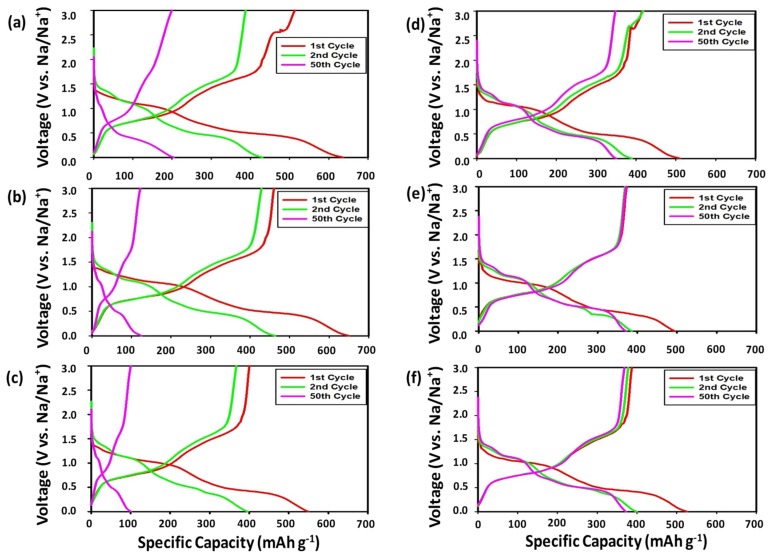
Voltage profiles of the SbTe-C30 electrode with (**a**–**c**) 0%, 2%, and 5% FEC (respectively) and the PVDF binder, and (**d**–**f**) 0%, 2%, and 5% FEC (respectively) and the PAA binder.

**Figure 5:**
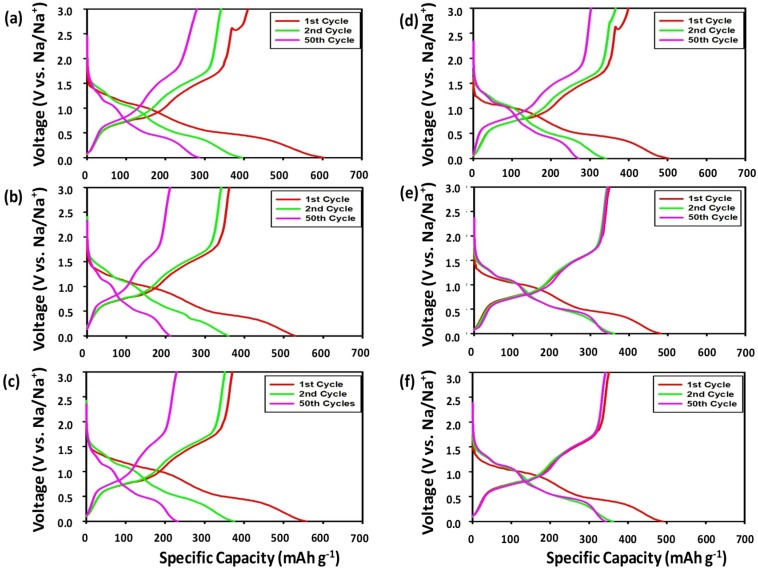
Voltage profiles of the SbTe-C40 electrode with (**a**–**c**) 0%, 2%, and 5% FEC (respectively) and the PVDF binder, and (**d**–**f**) 0%, 2%, and 5% FEC (respectively) and the PAA binder.

Figure 3, Figure 4 and Figure 5 display the voltage profiles of the SbTe-C20, SbTe-C30, and SbTe-C40 electrodes fabricated with the PVDF and PAA binders and with 0%, 2%, and 5% FEC added to the electrolyte. Each electrode was tested in the 0.0–3.0 V (vs. Na/Na^+^) voltage range at a current rate of 100 mA g^−1^. Figure 3 displays the voltage profiles of the SbTe-C20 electrode fabricated as described above. The electrode with the PVDF binder (Figure 3a–c) exhibits initial discharge and charge capacities of 648 mAh g^−1^ and 463 mAh g^−1^ at 0% FEC, 633 mAh g^−1^ and 467 mAh g^−1^ at 2% FEC, and 563 mAh g^−1^ and 426 mAh g^−1^ at 5% FEC, with initial coulombic efficiencies of 71%, 73%, and 75%, respectively, as listed in Table S1 (Supporting Information). However, no voltage plateaus were observed after 50 cycles, which indicates the loss of active material due to the pulverization induced by volume changes and particle aggregation, a result of the poor adhesion properties of the PVDF binder. On the other hand, the SbTe-C20 electrode prepared with the PAA binder (Figure 3e–f) exhibits initial discharge and charge capacities of 598 mAh g^−1^ and 431 mAh g^−1^ at 0% FEC, 553 mAh g^−1^ and 432 mAh g^−1^ at 2% FEC, and 539 mAh g^−1^ and 415 mAh g^−1^ at 5% FEC, with initial coulombic efficiencies of 72%, 78%, and 77%, respectively (Table S1, Supporting Information). Table S1 also reports the initial discharge and charge capacities, coulombic efficiencies, and capacity-retention percentages from the 2nd to the 100th cycle for the SbTe-C20 electrode. The data in Table S1 reveal that the cells with the PVDF binder show very poor capacity retentions, with 22.2%, 8.7%, and 8.1% of their capacities retained from the second to the 100th cycle for the 0%, 2%, and 5% FEC-containing electrolytes, respectively. On the other hand, the cells with PAA as the binder exhibited excellent capacity retentions of 65.7%, 96.2%, and 93.5% from the second to the 100th cycle for cells with the 0%, 2%, and 5% FEC-containing electrolytes, respectively. The initial irreversible capacities are due to electrolyte decomposition and the formation of solid electrolyte interphase (SEI) layers during the first desodiation/sodiation processes. The voltage plateaus in the discharge/charge traces overlap completely, which demonstrates that the PAA-incorporated electrodes exhibit excellent reversible kinetics even after 50 cycles. 

Figure 4 displays the voltage profiles of the SbTe-C30 electrodes fabricated with the PVDF and PAA binders and with 0%, 2%, and 5% FEC added to the electrolyte. The SbTe-C30 electrode with the PVDF binder (Figure 4a–c) exhibits initial discharge and charge capacities of 636 mAh g^−1^ and 513 mAh g^−1^ at 0% FEC, 650 mAh g^−1^ and 460 mAh g^−1^ at 2% FEC, and 549 mAh g^−1^ and 400 mAh g^−1^ at 5% FEC, with initial coulombic efficiencies of 80%, 70%, and 72%, respectively. The discharge/charge traces exhibit small voltage plateaus even after 50 cycles that are attributable to the increased carbon content, which buffers against volume expansion and maintains electrically conductive pathways, thereby minimizing the pulverization and aggregation of the active material. The SbTe-C30 electrode prepared with the PAA binder (Figure 4d–f) exhibits initial discharge and charge capacities of 510 mAh g^−1^ and 415 mAh g^−1^ at 0% FEC, 494 mAh g^−1^ and 373 mAh g^−1^ at 2% FEC, and 526 mAh g^−1^ and 387 mAh g^−1^ at 5% FEC, with initial coulombic efficiencies of 81%, 75%, and 73%, respectively. The voltage plateaus in the discharge/charge traces overlap completely, which indicates that the PAA-incorporated electrodes exhibit excellent reversible kinetics even after 50 cycles. Table S2 (Supporting Information) clearly reveals that increasing the carbon content increases the capacity retention capabilities of the cells with the PVDF binder, with 23.2%, 17.9%, and 17.4% capacity retentions observed from the second to the 100th cycle for the 0%, 2%, and 5% FEC-containing cells, respectively. However, the cells prepared with the PAA binder showed superior capacity retentions, with 95% and 93.4% retentions observed from the second to the 100th cycle for the 2% and 5% FEC-containing cells, respectively, which highlights the superior performance-enhancing ability of the PAA functional polymeric binder. The cell fabricated with PAA as the binder and 0% FEC lost almost all of its capacity due to electrode pulverization resulting from the unstable kinetics associated with the lack of FEC. 

Figure 5 displays the voltage profiles of the SbTe-C40 electrodes fabricated with the PVDF and PAA binders and 0%, 2%, and 5% FEC added to the electrolyte. The electrode prepared with the PVDF binder (Figure 5a–c) exhibits initial discharge and charge capacities of 603 mAh g^−1^ and 410 mAh g^−1^ at 0% FEC, 529 mAh g^−1^ and 361 mAh g^−1^ at 2% FEC, and 557 mAh g^−1^ and 370 mAh g^−1^ at 5% FEC, with initial coulombic efficiencies of 68%, 68%, and 66%, respectively. The reaction plateaus in the discharge/charge profiles of the SbTe electrode with 40% carbon are more prominent after 50 cycles, when compared to those of the SbTe electrode with 30% carbon, which indicates that increasing the carbon percentage helps to conserve the material and increase the capacity retention of the electrode. The SbTe-C40 electrode with the PAA binder (Figure 5d–f) exhibits initial discharge and charge capacities of 502 mAh g^−1^ and 399 mAh g^−1^ at 0% FEC, 482 mAh g^−1^ and 349 mAh g^−1^ at 2% FEC, and 488 mAh g^−1^ and 350 mAh g^−1^ at 5% FEC, with initial coulombic efficiencies of 79%, 72%, and 71%, respectively. The voltage plateaus in the discharge/charge traces overlap completely, which demonstrates the excellent reversible kinetics of the PAA-incorporated electrodes even after 50 cycles. Table S3 (Supporting Information) clearly demonstrates the positive effects of increase in carbon content with the cells exhibiting enhanced retention of capabilities. The cells with PVDF binder exhibit 40.5%, 42.6%, and 44.8% capacity retention from 2nd to 100th cycle for 0%, 2%, and 5% FEC, respectively. More enhanced retention capabilities were observed for the cells with PAA binder of 71.8%, 90.5%, and 94.4% capacity retention from 2nd to 100th cycle for 0%, 2%, and 5% FEC, respectively. While a lack of FEC renders both the PVDF-and PAA-based electrodes unstable for cycling, the addition of FEC clearly enhances electrode stability, which is attributable to the film-forming abilities and passivation capabilities of the FEC-containing electrolyte [35]. A clear trend with respect to carbon percentage is observed when the voltage profiles are analyzed; increasing the carbon content increases the reversibility of the electrode by accommodating volume changes, maintaining electrical pathways, and reducing active-material aggregation, which mitigates against the pulverization of the active material in the electrodes fabricated with the PVDF binder. On the other hand, increasing the carbon content of the PAA-based electrodes failed to produce any significant change apart from a reduction in capacity with increasing carbon content, due to lower amounts of loaded active material. The overwhelming effect of the PAA binder, which shadows the effect of the added carbon, is attributable to the superior adhesion capabilities of the PAA binder, which forms weak covalent bonds with the–OH groups on the surfaces of the active material and the current collector, which keeps the electrode intact [26,27].

#### 3.2.2. Differential Capacities 

**Figure 6:**
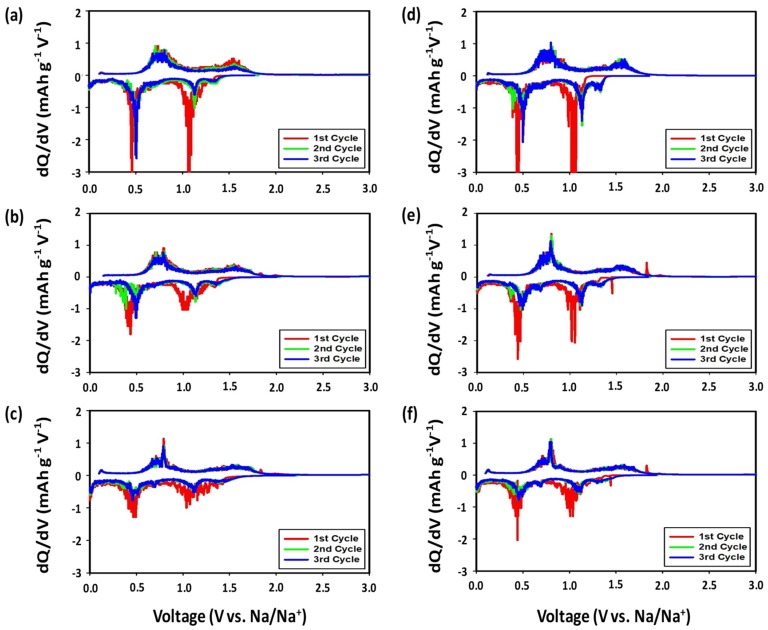
Differential capacity plots of the (**a**,**d**) SbTe-C20; (**b**,**e**) SbTe-C30; and (**c**,**f**) SbTe-C40 electrodes fabricated with (a–c) the PVDF binder with 5% FEC, and (d–f) the PAA binder with 5% FEC.

Figure 6 shows differential capacity (DCP) plots between 0.0 and −3.0 V over initial three cycles at a scan rate of 0.1 mV s^−1^ for the SbTe-C20, SbTe-C30, and SbTe-C40 electrodes prepared with the PVDF and PAA binders and 5% FEC added to the electrolyte. The first negative scans show sodiation peaks at around 1.4 V, 1.0 V, and 0.4 V, and two broad desodiation peaks at 0.5–0.8 V and 1.4–1.9 V (vs. Na^+^/Na). The sodiation peaks shift to the slightly higher potentials of 1.1 V and 0.5 V in the second and third scans for the electrodes fabricated with the PAA binder, while the potential differences between the sodiation and desodiation peaks decrease, which indicates reduced polarization. In addition, consecutive scans reveal peaks that overlap completely for the PAA-based electrodes, indicating excellent reversible kinetics. On the other hand, the second and third scans do not completely overlap for the SbTe-C20 and SbTe-C30 electrodes fabricated with the PVDF binder, while complete overlap is observed for the SbTe-C40 electrode, which highlights the performance-enhancing effect of the carbon buffer matrix. The small irreversible peak observed at 1.4 V in each first negative scan is due to initial processes associated with SEI formation, electrolyte decomposition, and Na-ion intercalation into the SbTe layer to form Na*_x_*SbTe. The peak at around 1.1 V is due to the transformation of Na*_x_*SbTe into Na_2_Te and Sb, while the peak at 0.4 V corresponds to the alloying of Na with Sb to form Na_3_Sb. The two broad desodiation peaks at 0.5–0.8 V and 1.4–1.9 V are ascribable to the de-alloying of Na_3_Sb and the formation of SbTe, respectively [36]. These sodiation/desodiation peaks correspond to, and are in agreement with, the plateau regions observed in the discharge/charge traces. The overall mechanism is in agreement with that reported for bimetallic SbTe. 

#### 3.2.3. Cycling Performance

**Figure 7:**
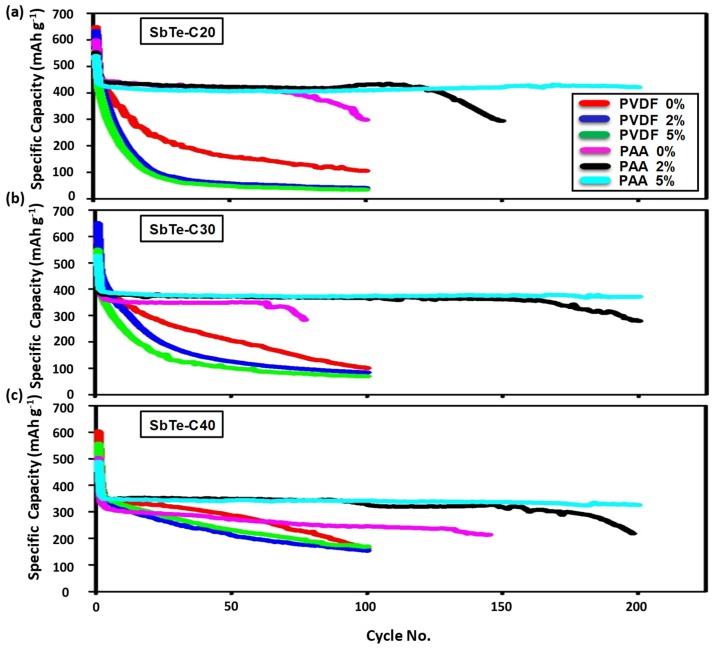
Cycling performance of the electrodes in this study prepared with the PVDF and PAA binders in cells with 0%, 2%, and 5% FEC added to the electrolyte: (**a**) SbTe-C20, (**b**) SbTe-C30, and (**c**) SbTe-C40.

Figure 7a–c display the cycling performance of SbTe-C20, SbTe-C30, and SbTe-C40 prepared with the PVDF and PAA binders and with 0%, 2%, and 5% FEC added to the electrolyte, respectively, with the data for SbTe-C20 shown in Figure 7a. The cells with the PVDF binder exhibit poor capacity retentions, with all cells losing most of their capacities within the first 50 cycles. On the other hand, the cells with the PAA binder show enhanced capacity retention capabilities, with the cell containing 2% FEC (Figure 7a) displaying excellent capacity retention for up to 100 cycles, after which the electrode becomes pulverized and rapidly loses its capacity, which we assume is due to the exhaustion of FEC through its decomposition to form NaF and vinylene carbonate (VC) [33] leading to cracks in the SEI and exposure of new active material and resulting in electrode degradation. It is noteworthy that the cell with 5% FEC exhibited excellent cyclability, with a discharge capacity of 421 mAh g^−1^ and a capacity retention of 96% after 200 cycles (Figure 7a). 

Figure 7b displays the cycling performance of the SbTe-C30 electrodes prepared with the PVDF and PAA binders and 0%, 2%, and 5% FEC added to the electrolyte. The cells with the PVDF binder exhibit poor capacity retentions with all cells losing most of their capacities within the first 50 cycles. On the other hand, the cells with PAA binder show enhanced capacity retention capabilities, with the cells containing 2% and 5% FEC (Figure 7b) exhibiting enhanced cyclabilities and discharge capacities of 367 and 373 mAh g^−1^, respectively, for up to 100 cycles, with negligible capacity losses. However, on further cycling, the cell with 2% FEC lost its capacity while the cell with 5% FEC exhibited 371 mAh g^−1^ and retained 92.9% of its second cycle capacity after 200 cycles.

Figure 7c displays the cycling performance of SbTe-C40 prepared with the PVDF and PAA binders and 0%, 2%, and 5% FEC added to the electrolyte. The cells with PVDF binder exhibit relatively better capacity retentions when compared with the cells containing 20% and 30% carbon, with retentions of 40.5%, 42.6%, and 44.8% observed for cells with 0%, 2%, and 5% FEC added to the electrolyte, respectively, after 100 cycles. On the other hand, the cells with the PAA binder showed enhanced capacity retention capabilities, with the cell containing 5% FEC (Figure 7c) exhibiting a discharge capacity of 325 mAh g^−1^ and a capacity retention of 90% over 200 cycles.

When the cycling performance of the SbTe-C20, SbTe-C30, and SbTe-C40 cells is analyzed, the electrodes with the PVDF binder and 5% FEC showed improved capacity retentions with increasing carbon percentage, with reversible capacities of 34 mAh g^−1^, 69 mAh g^−1^, and 168 mAh g^−1^ observed for the cells with 20%, 30%, and 40% carbon; these cells retained 8.1%, 17.4%, and 44.8% of their respective capacities after 100 cycles. Despite this, increasing the carbon contents of the cells prepared with the PAA binder has no apparent positive effect on cyclability or retention capability. The superior film-forming abilities and enhanced passivation capabilities of the FEC additive is also clearly observed in the cells devoid of the FEC additive, which are very unstable as evidenced by the peaks in their charge plots, whereas cells prepared with the FEC additive were stable. In addition, the cyclabilities and retention capabilities of the electrodes increased as the FEC percentage was increased from 2% to 5% when PAA was used as the binder. The cells with 2% FEC exhibited excellent cyclabilities for up to 100 cycles for all electrodes, whereas the cells with 5% FEC exhibited excellent cyclabilities for up to 200 cycles, as shown in Figure 7. These observations are attributable to the ability of FEC to prevent cracks resulting from binder degradation by providing negatively charged fluoride ions that react with Na^+^ ions to form NaF; FEC decomposes into NaF and VC, and VC further decomposes to release CO_2_ which forms Na_2_CO_3_, a favorable SEI component that results in an improved SEI layer [33].

#### 3.2.4. Rate Capabilities and Capacity Retentions

**Figure 8:**
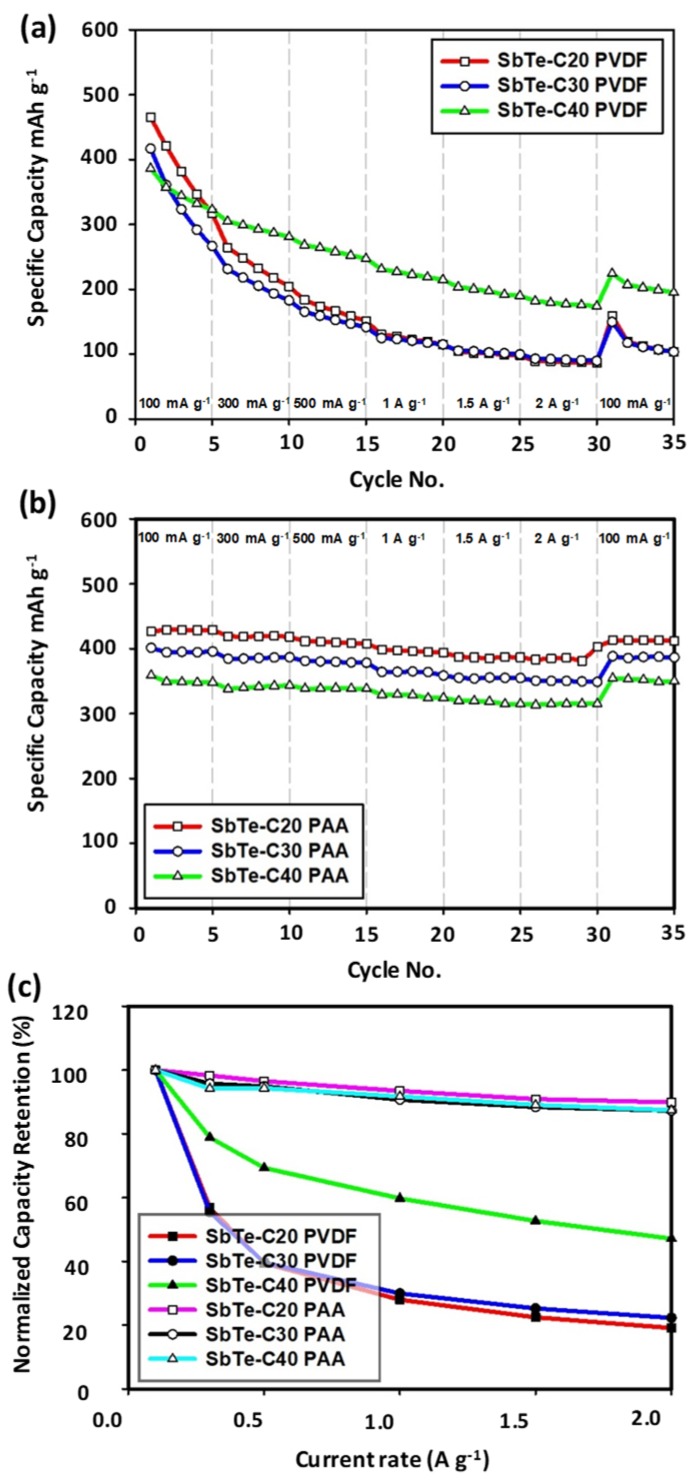
Rate capabilities of the SbTe-C20, SbTe-C30, and SbTe-C40 electrodes with 5% FEC added to the electrolyte and (**a**) PVDF as the binder and (**b**) PAA as the binder. (**c**) Normalized charge-capacity retentions as functions of current density.

Figure 8a,b show the rate capabilities of the SbTe-C20, SbTe-C30, and SbTe-C40 electrodes prepared with the PVDF and PAA binders and 5% FEC added to the electrolyte. The cells were cycled five times at various current densities, namely 100 mA g^−1^, 300 mA g^−1^, 500 mA g^−1^, 1 A g^−1^, 1.5 A g^−1^, and 2 A g^−1^, and 100 mA g^−1^. The reversible charge capacities of the cells prepared with the PVDF binder and 5% FEC were found to be 465, 264, 183, 130, 104, 88, and 159 mAh g^−1^ for SbTe-C20; 416, 231, 165, 124, 105, 92, and 149 mAh g^−1^ for SbTe-C30; and 386, 304, 267, 230, 203, 181, and 224 mAh g^−1^ for SbTe-C40, respectively, at the above-mentioned current densities, with SbTe-C20, SbTe-C30, and SbTe-C40 exhibiting capacity retentions of 34%, 36%, and 58%, respectively, when the current density was returned to 100 mA g^−1^. On the other hand, the cells prepared with PAA as the binder and 5% FEC exhibited reversible charge capacities of 426, 419, 411, 398, 387, 383, and 413 mAh g^−1^ for SbTe-C20; 401, 384, 381, 364, 355, 350, and 388 mAh g^−1^ for SbTe-C30; and 358, 338, 338, 329, 319, 313, and 354 mAh g^−1^ for SbTe-C40, at the above mentioned current densities, respectively, with SbTe-C20, SbTe-C30, and SbTe-C40 exhibiting capacity retentions of 97%, 97%, and 99%, respectively, when the current density was returned to 100 mAh g^−1^. Figure 8c displays the normalized charge-capacity retentions at current densities between 0.1 and 2 A g^−1^ for the cells containing 5% FEC in the electrolyte and PVDF and PAA as binders. The retention plots for the PVDF-based cells show retention rates that increase with increasing carbon percentage, with SbTe-C40 showing a retention of 47% at a current density of 2A g^−1^, which clearly demonstrates the performance-enhancing effect of the carbon buffer matrix that encapsulates the active material, maintains conductive pathways, and mitigates against active-material isolation. However, increasing the carbon percentage in the cells prepared with PAA binder has a minuscule effect on capacity retention, with these cells exhibiting 89%, 87%, and 87% retentions at a current density of 2A g^−1^. These rate capabilities and normalized capacity retention data highlight the positive effect of carbon as a conductive buffer matrix, and the superior adhesion capabilities and performance-enhancing properties of PAA as a functional polymeric binder.

#### 3.2.5. Electrochemical Impedance Spectroscopy (EIS)

**Figure 9:**
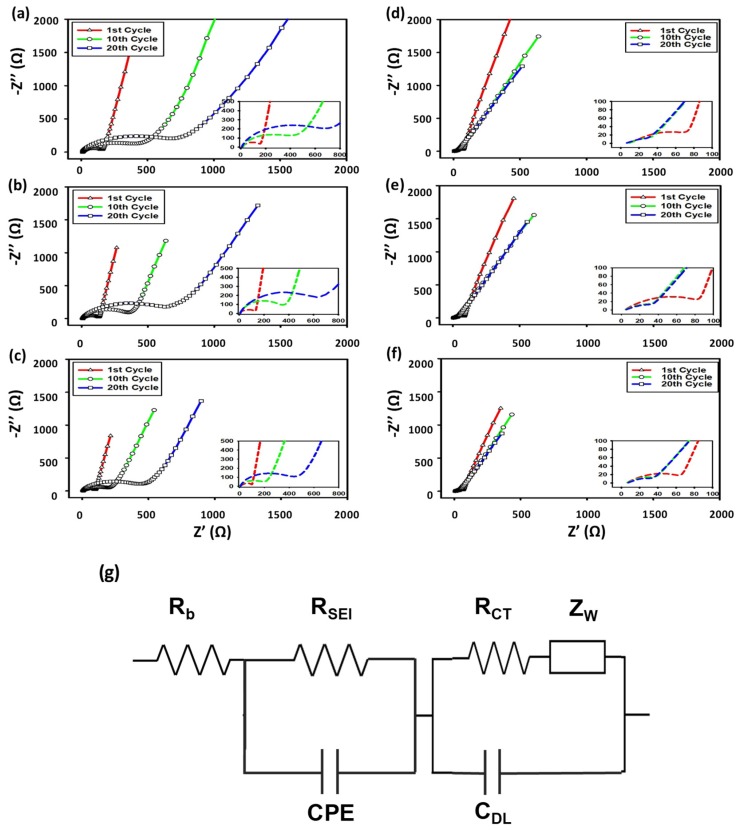
EIS-based Nyquist plots for the SbTe-C20, SbTe-C30, and SbTe-C40 electrodes in cells with 5% FEC added to the electrolyte: (**a**–**c**) PVDF binder and (**d**–**f**) PAA binder. (**g**) The equivalent circuit.

Figure 9 shows electrochemical impedance spectra collected at the 1st, 10th, and 20th cycles for the SbTe-C20, SbTe-C30, and SbTe-C40 electrodes prepared with the PVDF and PAA binders, in cells with 5% FEC added to the electrolyte. The simplified equivalent circuit is shown Figure 9g, in which R_b_ is attributed to the electrolyte resistance, R_SEI_ is attributed to the SEI layer resistance, and R_CT_ is attributed to the charge-transfer resistance, while C_DL_ is the interfacial double layer capacitance, CPE is the constant phase element, and Z_W_ is the Warburg impedance. The EIS-based Nyquist plots exhibit compressed semicircles in the mid frequency region that correspond to the charge-transfer resistance (R_CT_) at the electrode–electrolyte interface. The sloping lines in the low frequency region corresponds to Na^+^ diffusion into the electrode [31,37,38]. The EIS spectra of the cells with the electrodes prepared with the PVDF binder and 5% FEC added to the electrolyte (Figure 9a–c) show huge increases in the sizes of the semicircles corresponding to the charge-transfer resistance (R_ct_) after the 1st, 10th, and 20th cycles, with the semicircle diameters increasing with increasing numbers of cycles. This increase in R_ct_ is ascribable to the isolation of active material as cracks are formed due to the volume changes associated with desodiation/sodiation [39]. The poor binding ability of PVDF leads to the collapse and cracking of the electrode during cycling, which leads to higher charge-transfer resistances (R_CT_). The insets in the figures provide expanded views of the Nyquist plots that reveal (upon closer inspection) the effect of the carbon percentage in the electrodes with the PVDF binder, with R_CT_ observed to decrease with increasing carbon percentage. This ascribable to the carbon support matrix, which encapsulates the active material, buffers volume expansions, and maintains conductive pathways, thereby preventing cracking and the segregation of the active material [16]. On the other hand, the cells containing the electrodes with PAA binder and 5% FEC added to the electrolyte (Figure 9d–f) show decreases in charge-transfer resistance with increasing cycle number, with the semicircle diameters observed to decrease. We observed that the Nyquist plots at the 10th and 20th cycle almost completely overlap, highlighting the superior binding capability of the PAA binder, which accommodates volume expansions and contractions and keeps the electrodes intact. The progressively decreasing low impedance values of the electrodes prepared with the PAA binder demonstrate that the functional PAA binder uniformly coats the surfaces of the particles to form ionic conductive films that stabilize SEI layers [31], thereby engendering the electrodes with excellent electrochemical performance and enhanced cycling stabilities.

### 3.3. Ex Situ SEM

**Figure 10:**
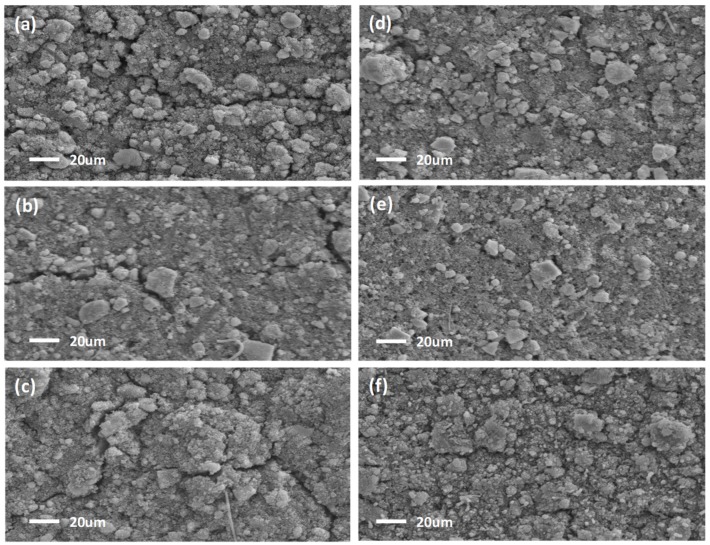
Postmortem SEM images of the (**a**,**d**) SbTe-C20, (**b**,**e**) SbTe-C30, and (**c**,**f**) SbTe-C40 electrodes prepared with (**a**–**c**) the PVDF binder and (**d**–**f**) the PAA binder after cycling in cells with 5% FEC added to the electrolyte.

Ex situ SEM images of the cycled SbTe-C20 (Figure 10a,d), SbTe-C30 (Figure 10b,e), and SbTe-C40 (Figure 10c,f) electrodes prepared with the PVDF and PAA binders cycled in cells with 5% FEC added to the electrolyte, are displayed in Figure 10. The electrodes were extracted from cycled cells inside an argon-filled glove box and rinsed with propylene carbonate to remove any excess residual electrolyte salts, the electrodes were then dried in a glove box. The surface morphologies of the samples were examined by ex situ SEM. The electrodes prepared with the PVDF binder exhibit large continuous cracks across their surfaces, while the electrodes prepared with the PAA binder are devoid of any visible cracks. The cracks are the result of the poor adhesion properties of the PVDF binder, whereas the superior adhesion capability of the PAA binder keeps the electrode intact by withstanding volume expansions. The PVDF-based electrodes also show surface fragments and aggregated spherical particles. Overall the surfaces of the PVDF-based electrodes appear to be rough when compared to those of the PAA-based electrodes, which is ascribable to the decomposition of the electrolyte during prolonged cycling [39]. The electrodes prepared with the PAA binder are comparatively less rough due to the thin SEI layers formed as a result of the conductive ionic films on the surface of active material formed by PAA binder. The thin SEI layer enhances ion transport into each electrode by reducing the charge-transfer resistance [39], which is in agreement with the impedance results obtained for the PAA-based electrodes. These observations explain the long cyclabilities and capacity retention capabilities of the PAA-based electrodes, which highlight the superior adhesion properties of the PAA binder that keeps the electrode intact and adheres the active material to the current collector. Numerous surface fragments and spherical agglomerated particles were observed on the surfaces of the electrodes prepared with 20% carbon (Figure 10a,d), which decrease in number with increasing carbon content, with the 40%-carbon-containing electrode exhibiting a much more uniform surface. The agglomerations are due to the aggregation of active material upon prolonged cycling. The excess carbon mitigates against active-material aggregation by uniformly coating the surface of the active material and providing electrically conducting pathways.

## 4. Conclusions

In this work, we synthesized SbTe bimetallic compounds containing 20%, 30%, and 40% carbon support matrices through a scalable and easy to perform HEMM process. These materials were examined as potential SIB anodes with two different binders, namely PVDF and PAA. The cells containing PAA as the binder exhibited excellent electrochemical performance, and were significantly superior when compared to the cells containing the PVDF binder. Although increasing the carbon percentage resulted in enhanced cyclabilities and capacity retentions in cells with the PVDF binder, they still performed poorly, with all cells losing most of their capacities within the first 50 cycles. On the other hand, the cells with the PAA binder showed excellent capacity retentions and cycling stabilities; the SbTe-C20 electrode with 5% FEC added to the electrolyte exhibited an excellent reversible capacity of 421 mAh g^−1^ after 200 cycles. Increasing the carbon percentage increases the cycling stability of the cell, with SbTe-C30 and SbTe-C40 exhibiting 371 mAh g^−1^ and 325 mAh g^−1^ after 200 cycles, respectively. The carbon support matrix plays a significant role in improving the stability, cyclability, and capacity retention of the electrode. However, when the tradeoff between capacity and the cyclability associated with increasing carbon percentage is considered, the binder plays a significantly more prominent role in obtaining higher capacities, enhanced cyclabilities, and superior rate capabilities.

## Supplementary Materials

The following are available online at https://www.mdpi.com/2079-4991/9/8/1134/s1, Figure S1: SEM images of the SbTe-C30 electrode prepared with (a) the PVDF binder and (e) the PAA binder. Sb, Te, and C EDS maps corresponding to: (b)–(d) panel a, and (f)–(h) panel e, Figure S2: SEM images of the SbTe-C40 electrode prepared with (a) the PVDF binder and (e) the PAA binder. Sb, Te, and C EDS maps corresponding to: (b)–(d) panel a, and (f)–(h) panel e, Table S1: Electrochemical data of SbTe-C20 electrodes, Table S2: Electrochemical data of SbTe-C30 electrodes, Table S3: Electrochemical data of SbTe-C40 electrodes.
