# Supplementary material for: Enhancing the Electrochemical Performance of SbTe Bimetallic Anodes for High-Performance Sodium-Ion Batteries: Roles of the Binder and Carbon Support Matrix
Source: Nanomaterials (Basel). 2019 Aug 7;9(8):1134. doi: 10.3390/nano9081134 (PMC6723861; doi:10.3390/nano9081134)
Supplement: Supplementary file 1 [file nanomaterials-09-01134-s001.pdf]

Supplementary Materials

Enhancing the Electrochemical Performance of SbTe Bimetallic Anodes for High-Performance Sodium-Ion Batteries: Roles of the Binder and Carbon Support Matrix

Vijay Mohan Nagulapati, Doo Soo Kim, Jinwoo Oh, Jin Hong Lee, Jaehyun Hur,

Il Tae Kim, and Seung Geol Lee

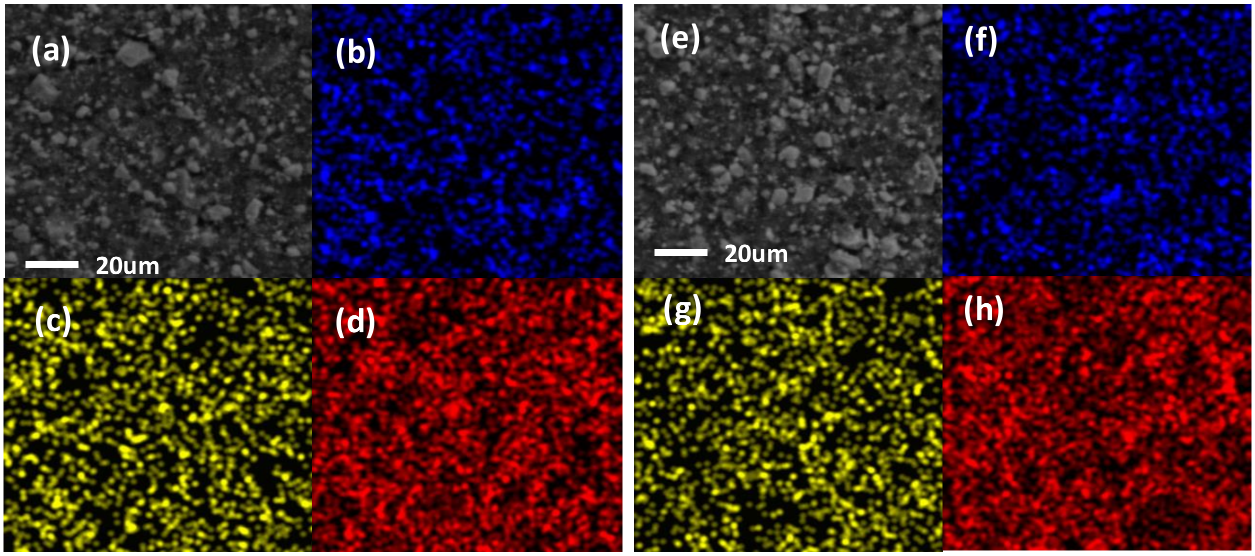

Figure 1. SEM images of the SbTe-C30 electrode prepared with (**a**) the PVDF binder and (**e**) the PAA binder. Sb, Te, and C EDS maps corresponding to: (**b**)–(**d**) panel a, and (**f**)–(**h**) panel e.

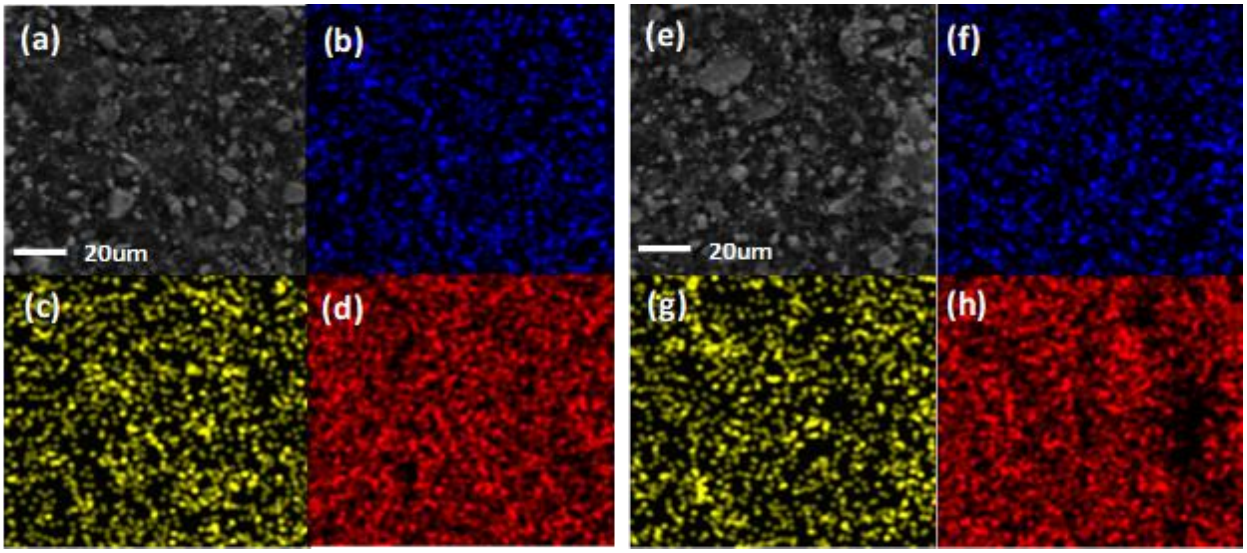

Figure S2. SEM images of the SbTe-C40 electrode prepared with (**a**) the PVDF binder and (**e**) the PAA binder. Sb, Te, and C EDS maps corresponding to: (**b**)–(**d**) panel a, and (**f**)–(**h**) panel e**.**

Table S1. Electrochemical data of SbTe-C20 electrodes.

Binder and FEC %	1st cycle discharge capacity (mAh g^{-1})	1st cycle charge capacity (mAh g^{-1})	1st cycle Coulombic efficiency (%)	2nd cycle discharge capacity (mAh g^{-1})	100th cycle discharge capacity (mAh g^{-1})	capacity retention 2nd to 100th cycle (%)
PVDF & 0% FEC	648	463	71	471	105	22.2
PVDF & 2% FEC	633	467	73	466	41	8.7
PVDF & 5% FEC	563	426	75	417	34	8.1
PAA & 0% FEC	598	431	72	453	298	65.7
PAA & 2% FEC	553	432	78	448	431	96.2
PAA & 5% FEC	539	415	77	436	408	93.5

Table S2. Electrochemical data of SbTe-C30 electrodes.

Binder and FEC %	1st cycle discharge capacity (mAh g–1)	1st cycle charge capacity (mAh g–1)	1st cycle Coulombic efficiency (%)	2nd cycle discharge capacity (mAh g–1)	100th cycle discharge capacity (mAh g–1)	capacity retention 2nd to 100th cycle (%)
PVDF & 0% FEC	636	513	80	430	100	23.2
PVDF & 2% FEC	650	460	70	463	83	17.9
PVDF & 5% FEC	549	400	72	396	69	17.4
PAA & 0% FEC	510	415	81	389	1.1	-
PAA & 2% FEC	494	373	75	386	367	95
PAA & 5% FEC	526	387	73	399	373	93.4

Table S3. Electrochemical data of SbTe-C40 electrodes.

Binder and FEC %	1st cycle discharge capacity (mAh g^{-1})	1st cycle charge capacity (mAh g^{-1})	1st cycle Coulombic efficiency (%)	2nd cycle discharge capacity (mAh g^{-1})	100th cycle discharge capacity (mAh g^{-1})	capacity retention 2nd to 100th cycle (%)
PVDF & 0% FEC	603	410	68	395	160	40.5
PVDF & 2% FEC	529	361	68	359	153	42.6
PVDF & 5% FEC	557	370	66	375	168	44.8
PAA & 0% FEC	502	399	79	341	245	71.8
PAA & 2% FEC	482	349	72	361	327	90.5
PAA & 5% FEC	488	350	71	361	341	94.4
